# Association of thiazolidinediones with gastric cancer in type 2 diabetes mellitus: a population-based case–control study

**DOI:** 10.1186/1471-2407-13-420

**Published:** 2013-09-17

**Authors:** Shen-Shong Chang, Hsiao-Yun Hu

**Affiliations:** 1 Division of Gastroenterology, Taipei City Hospital Yang-Ming branch, Taipei, Taiwan; 2 Department of internal Medicine, Taipei City Hospital Yang-Ming branch, Taipei, Taiwan; 3 School of Medicine, National Yang-Ming University, Taipei, Taiwan; 4 Institute of Public Health & Department of Public Health, National Yang-Ming University, Taipei, Taiwan; 5 Department of Education and Research, Taipei City Hospital, Taipei, Taiwan

**Keywords:** Peroxisome proliferator-activated receptors, Thiazolidinediones, Gastric cancer, Case–control, Diabetes mellitus

## Abstract

**Background:**

It has been shown that peroxisome proliferator-activated receptors (PPAR) have physiological and pharmacological ligands. The objective is to assess the association between thiazolidinediones (TZDs) and the occurrence of gastric cancer.

**Methods:**

We conducted a population-based nested case–control study. Data were retrospectively collected from the National Health Insurance Research Database (NHIRD). The cases consisted of all diabetes mellitus (DM) patients aged 30 to 99 years, and who had a first time diagnosis of gastric cancer in the study cohort. The controls were matched to cases by age, sex, and index date. The adjusted odds ratio (OR) and 95% confidence interval (CI) were estimated by using multiple logistic regression.

**Results:**

Records from 357 gastric cancer and 1,428 selected matched controls were included in the analyses of gastric cancer risk. A total of 7% or 9.5% of the cases and 10.8% or 14.8% of the controls had used any quantity of at least 2 prescriptions for pioglitazone or rosiglitazone, respectively. After adjusting for possible confounders, pioglitazone (OR = 0.93, P *>* 0.05) and rosiglitazone (OR = 1.21, P *>* 0.05), had no significant association of decreasing gastric cancer. After adjusting for possible confounders, pioglitazone (OR = 0.70, P *>* 0.05) or rosiglitazone (OR = 0.79, P *>* 0.05), had no significant trend toward decreasing gastric cancer risk with increasing cumulative doses ≥ 260 defined daily doses (DDDs), respectively. Moreover, adjusting for possible confounders pioglitazone (OR = 0.68, P *>* 0.05) or rosiglitazone (OR = 0.74, P *>* 0.05) had no significant trend toward decreasing gastric cancer risk with increasing cumulative doses ≥ 1 year, respectively.

**Conclusions:**

Our results did not show evidence to support that TZD derivatives in DM patients reduces gastric cancer occurrence.

## Background

Gastric carcinoma is the second most common cancer in the world [1]. The diagnosis of cancer and diabetes in the same people occurs more frequently [2]. Numerous factors may affect the positive association between diabetes and cancer. Potential risk factors common to both diseases include age, sex, physical activity, obesity, diet, alcohol, and smoking [3-6]. Numerous studies have been performed to research therapeutic targets and drugs capable of preventing and treating gastric carcinoma and other malignancies. Evidence from observational studies shows that oral hypoglycemic agents are associated with either an increased or reduced risk of cancer [7].

Peroxisome proliferator-activated receptors (PPARs) indicate a family of nuclear receptors that are related to thyroid hormones, insulin sensitivity, adipocyte differentiation, and retinoid receptors [8,9]. Three PPAR subtypes, PPAR-α、β、γ have been indentified. It has been shown that PPAR-γ has physiological and pharmacological ligands [9]. Anti-diabetic thiazolidinediones (TZDs), such as pioglitazone and rosiglitazone, belong to synthetic PPAR-γ, which can decrease insulin resistance in peripheral tissues and hepatocytes, and increase the action of insulin hormones [10]. PPAR-γ is implicated as a putative therapeutic target for cancer in a variety of tumors as several observations have suggested that stimulation of PPAR-γ function may inhibit carcinogenesis and tumor cell growth [11,12]. Ligands of PPAR-γ have been demonstrated to suppress the propagation of these cancers in vitro [13-16]. One well-known category of ligands is the TZDs, which include rosiglitazone and 15-deoxy-prostaglandin-J2 (15d-PGJ(2)) [17]. Lu et al. [18] has previously reported that troglitazone suppresses stomach cancer through the activation of PPAR-γ. It had been reported that stomach cancer is suppressed by PPAR-γ-ligand-mediated apoptosis [19].

Konturek et al. [20] has recently shown that PPAR-γ is implicated in *Helicobacter pylori (H. pylori)*-related gastric carcinogenesis, and that a PPAR-γ agonist may have potential in a therapeutic cancer role. In contrast to the link between the PPAR-γ ligand and gastric carcinoma in vitro study, results of other clinical studies remain unknown.

No large clinical trial or nationally representative observational study has been conducted to address this issue. Thus, we conducted a nested case–control study based on the National Health Insurance Research Database (NHIRD) in Taiwan. The main outcome of interest is to assess the association between TZDs (pioglitazone and rosiglitazone) and the occurrence of gastric cancer.

## Methods

### Data source

This nationwide cohort study was based on patient data obtained from the National Health Insurance Database (NHID), which is managed by the Taiwan National Health Research Institute (NHRI). The NHID contains health care data for 99% of the population of Taiwan (approximately 23 million people). The NHI sample files, which are established and managed by the NHRI, consist of comprehensive use and enrollment information for a randomly selected sample of 1,000,000 NHI beneficiaries, representing approximately 5% of all enrollees in Taiwan in 2000. The NHRI is the only institute that is approved to conduct samplings of a representative portion of the entire population. Although privacy protections are maintained, the reimbursement data for sampled patients were retrieved and used for academic research after obtaining approval. The NHID contains comprehensive information, including demographic data, dates of clinical visits, diagnostic codes, and details of prescriptions. The International Classifications of Diseases, Revision 9, Clinical Modification (ICD-9-CM) was used to define diseases during the period of this study. This study has been approved by the NHRI.

### Study design

A nested case–control approach is a useful alternative to cohort analysis to study time-dependent exposure [21]. The risk estimates from cohort and nested case–control analysis should be similar if confounding factors are controlled in both analyses. The strength of the nested case–control study design may be particularly useful in rare cases [22].

### Study patients

Diabetes mellitus (DM) patients were identified using inpatient discharge records, or by 3 or more ambulatory care claims with a diagnosis of ICD-9-CM: 250. From the NHID, patients who had DM, and were using pioglitazone and rosiglitazone between January 1, 2000 and December 31, 2010 were compared to DM patients who were not treated with pioglitazone or rosiglitazone. Patients who had ever received a gastrectomy or vagotomy were excluded from the analyses. Patients with a previous diagnosis of gastric cancer or Zollinger–Ellison syndrome, and those who were less than 30 years old and more than 99 years old were also excluded. We further excluded those who had a hospital admission with a discharge diagnosis of insulin-dependent diabetes mellitus (ICD-9-CM 250.x1, 250.x3) or received a catastrophic illness certificate issued by the Department of Health for Type 1 Diabetes.

### Exposure to pioglitazone or rosiglitazone

Information on all TZDs prescription was extracted from the NHRI prescription database. The defined daily dose (DDD) is the assumed average maintenance dose per day for drugs administered to adults and used according to their main indications. The DDDs recommended by the World Health Organization (WHO) [23] were used to quantify the use of TZDs. Cumulative DDD was estimated as the sum of dispensed DDDs of any TZDs (pioglitazone or rosiglitazone) from January 1, 2000 to the index date. The gathered data comprised the date of prescription, daily dosage, and the number of days of drug supply. The main exposure of interest was the use of pioglitazone or rosiglitazone, which entered Taiwan’s market in June 2001 and March 2000, respectively.

### Definition of gastric cancer

All patients aged 30–99 years in the study cohort, with the first occurrence of stomach cancer ICD-9-CM 150.0-150.9 during the 11-year period, were included as cases based on inpatient discharge records. Patients with a previous diagnosis of gastric cancer were excluded. A diagnosis of gastric cancer in the NHID required histologic confirmation to be reported to the registry of the Catastrophic Illness Patient Database. All potential cases were validated by a linkage through the National Cancer Registry.

### Definition of control group

A risk-set sample (control sample from those in the original study cohort who remained free of outcome at the time when a case occurred) matched by age (within 5 years), sex, and the number of days of follow-up, was used as controls for the cohort. For newly diagnosed Type 2 Diabetes patients, case and controls were also matched based on antidiabetic treatment duration (within 30 days) at cancer diagnosis. For newly diagnosed diabetic patients, the scheme that matched follow-up duration also considered diabetic duration. For prevalent patients with unknown duration, we selected controls with the same follow-up duration to reduce the confounding effect of diabetes duration. Up to four controls were selected for each patient [24].

### Definition of peptic ulcer history and ulcer bleeding history

All endoscopically-diagnosed peptic ulcers in DM patients prior to the date of gastric cancer diagnosis, according to ambulatory care and inpatient discharge records, were used for peptic ulcer history. Peptic ulcers were defined as gastric ulcers (ICD-9-CM 531), duodenal ulcers (ICD-9-CM 532), and nonspecific peptic ulcers (ICD-9-CM 533) following endoscopic confirmation from January 1, 2000 to the index date. Based on inpatient discharge records prior to the date of gastric cancer diagnosis, peptic ulcer bleeding (following endoscopic confirmation) was used as ulcer bleeding history. Peptic ulcer bleeding was defined using ICD-9-CM codes 531.0, 531.2, 531.4, 531.6, 532.0, 532.2, 532.4, 532.6, 533.0 533.2, 533.4 and 533.6 following endoscopic confirmation from January 1, 2000 to the index date.

### Definition *H. pylori* eradication rate

Patients placed into the category of *H. pylori* eradication therapy were defined as those who received triple or quadruple therapy during the same inpatient discharge record or outpatient visit from January 1, 2000 to the index date. The duration of therapy was between 7 and 14 days. *H. pylori* infection is treated with multidrug regimen that consists of proton pump inhibitors (PPIs) or histamine receptor-2 blockers (H_2_-blockers), clarithromycin or tetracycline, amoxicillin or metronidazole, and potentially bismuth. The PPIs administered to patients that were evaluated in this study were lansoprazole, esomeprazole, omeprazole, pantoprazole, and rabeprazole and the H_2_-blockers were cimetidine, famotidine, nizatidine, ranitidine, and roxatidine [25].

### Definition of comorbidities

Patient comorbidities were identified using inpatient discharge records or by 3 or more ambulatory care claims with the diagnosis of coronary artery disease (CAD): ICD-9-CM 410–414, cerebral vascular disease (CVD): ICD-9-CM 430–438, chronic liver disease (CLD): 070.2x, 070.3x, V02.61, 070.41, 070.44, 070.51, 070.54, V02.62, 571.4, 571.2, 571.5, 571.6, 571.0x, 571.1x, 571.2, and 571.3x, chronic obstructive pulmonary disease (COPD): ICD-9-CM 490–492, 494, and 496, chronic kidney disease (CKD): ICD-9-CM 580–589, 250.4, 274.1, 283.11, 403.x1, 404.x2, 404.x3, 440.1, 442.1, 447.3, 572.4, 642.1x, 646.2x, and 794.4, and gastroesophageal reflux disease (GERD): 530.81 or erosive esophagitis (EE): 530.11.

### Use of medication

Patients were categorized by their use of metformin, sulfonylurea, glucosidase inhibitors, meglitinides, dipeptidyl peptidase 4 (DPP-4) inhibitors, insulin, statins, angiotensin receptor blockers (ARBs), angiotensin-converting enzyme (ACE) inhibitors, aspirin, cyclooxygenase-2 (COX-2)-specific inhibitors, and non-steroidal anti-inflammatory drugs (NSAIDs) with at least 2 prescription prior to the index date of gastric cancer diagnosis [26].

### Statistical analysis

For comparisons of proportions, *χ*^2^ statistics were used. A conditional logistic regression model was used to estimate the relative magnitude in relation to the use of TZDs. Exposure was defined as patients who received at least 2 prescriptions for a TZD at any time between January 1, 2000 and the index date [26]. In the analysis, the participants were categorized into one of 2 TZDs exposure categories: nonuse, past use, and recent use. Furthermore, we divided the person-time-product into recent use (including current medication and discontinuation of medication prior to gastric cancer diagnosis < 6 months), past use (drug discontinuation to gastric cancer diagnosis ≥ 6 months), and non-use. The participants were categorized into users of dosages less than the median (< 260 DDDs) and users of dosages equal or greater than the median (≥ 260 DDDs). In the dose- and duration- response analysis, we calculated the odds ratios (OR) for higher (≥ 260 DDDs) or lower (< 260 DDDs), and for cumulative treatment duration ≥ 1 year or < 1 year. The OR and their 95% confidence intervals (CI) were calculated using patients with no exposure as the reference. All statistical analyses implemented in the present study were performed using an SAS statistical package (SAS System for Windows, version 9.2; SAS Institute, Cary, NC, USA).

## Results

Records from 357 gastric cancer and 1,428 selected matched controls were included in the analyses of gastric cancer risk. Table 1 presents the distribution of demographic characteristics, such as age, sex, peptic ulcer history, ulcer bleeding history, *H. pylori* eradication rate, comorbidities, and medication of gastric cancer cases and controls. The patients had significantly higher rates of peptic ulcer history and ulcer bleeding history. No significant difference between the patients and the controls was found for the *H. pylori* eradication rate.

**Table 1:** Characteristics, comorbidities, and medication use among cases and controls

**Variables**	**Cases**		**Controls**		**P value**
	**N = 357**	**%**	**N = 1,428**	**%**	
Age at DM					0.483
30-60	69	15.90	300	17.28	
≥60	288	66.36	1,128	64.98	
Sex					1.000
Male	215	49.54	568	32.72	
Female	142	32.72	860	49.54	
Peptic ulcer history	172	39.63	334	19.24	<0.001
Ulcer bleeding history	61	14.06	156	8.99	<0.001
HP eradication rate	33	7.60	139	8.01	0.779
Comorbidities					
CAD	120	27.65	603	34.74	0.003
CVD	84	19.35	466	26.84	<0.001
CLD	78	17.97	337	19.41	0.484
COPD	98	22.58	465	26.79	0.063
CKD	77	17.74	431	24.83	0.001
GERD or EE	21	4.84	91	5.24	0.733
Medications					
Pioglitazone	25	5.76	154	8.87	0.033
Rosiglitazone	34	7.83	211	12.15	0.010
Metformin	236	54.38	990	57.03	0.241
Sulfonylurea	256	58.99	1,022	58.87	0.958
Glucosidase inhibitors	36	8.29	293	16.88	<0.001
Meglitinides (Glinides)	35	8.06	241	13.88	<0.001
DPP-4 inhibitors	1	0.23	85	4.90	<0.001
Insulin	37	8.53	265	15.26	<0.001
Statins	86	19.82	531	30.59	<0.001
ARBs	88	20.28	534	30.76	<0.001
ACE inhibitors	126	29.03	560	32.26	0.173
Aspirin	118	27.19	610	35.14	<0.001
COX-2 inhibitors	27	6.22	189	10.89	0.003
NSAIDs	74	17.05	432	24.88	<0.001

*DM* diabetes mellitus, *HP Helicobacter pylori, CAD* coronary artery disease, *CVD* cerebral vascular disease, *CLD* chronic liver disease, *COPD* chronic obstructive pulmonary disease, *CKD* chronic kidney disease, *GERD* gastroesophageal reflux disease, *EE* erosive esophagitis, *DPP-4 inhibitors* dipeptidyl peptidase 4 inhibitors, *ARBs* angiotensin receptor blockers, *ACE inhibitors* angiotensin-converting enzyme inhibitors, *COX-2 inhibitors* cyclooxygenase-2 specific inhibitors, *NSAIDs* non-steroidal anti-inflammatory drugs. *N* number.

The relationship between the use of TZDs and gastric cancer is shown in Tables 2 and 3. A total of 7% of the patients and 10.8% of the controls had used at least 2 prescriptions for pioglitazone, as shown in Table 2. Any-use of pioglitazone (OR = 0.62, P < 0.05) was associated with a decreased crude OR for gastric cancer. However, after adjusting for possible confounders (including age, sex, peptic ulcer history, ulcer bleeding history, *H. pylori* eradication rate, comorbidities, and medication), any-use of pioglitazone (OR = 0.93, P *>* 0.05) had no significant association with decreasing gastric cancer. When pioglitazone use was categorized by cumulative dosage, the crude OR was 0.77 (P *>* 0.05) for the group with cumulative pioglitazone use < 260 DDDs, and was 0.49 (P < 0.05) for the group with cumulative pioglitazone use ≥ 260 DDDs, compared with non-use. After adjusting for possible confounders, no significant trend was observed toward decreasing gastric cancer risk with increasing cumulative doses ≥ 260 DDDs (OR = 0.70, P *>* 0.05). When pioglitazone use was categorized by cumulative duration, the crude OR was 0.73 (P *>* 0.05) for the group with cumulative pioglitazone use < 1 year, and was 0.47 (P < 0.05) for the group with cumulative pioglitazone use ≥1 year, compared with non-use. After adjusting for possible confounders, no significant trend was observed toward decreasing gastric cancer risk with increasing cumulative duration ≥1 year (OR = 0.68, P *>* 0.05), as shown in Table 2.

**Table 2:** Associations between pioglitazone use and gastric cancer risk in a population-based nested case–control study

**Variables**	**Pioglitazone**						
	**Cases**		**Controls**		**Crude OR**	^**†**^**Adjusted OR**	^**‡**^**Adjusted OR**
	**N = 357**	**%**	**N = 1,428**	**%**			
Nonuse	332	93.0	1,274	89.2	1.00	1.00	1.00
Any use	25	7.0	154	10.8	0.62*	0.65*	0.93
Recent use	11	3.1	75	5.3	0.56*	0.54	0.70
Past use	14	3.9	79	5.5	0.68*	0.77	1.28
Cumulative dosage							
< 260 DDDs	15	4.2	75	5.3	0.77	0.78	1.19
≥ 260 DDDs	10	2.8	79	5.5	0.49*	0.52	0.70
Cumulative duration							
< 1 year	17	4.8	89	6.2	0.73	0.74	1.14
≥ 1 year	8	2.2	65	4.6	0.47*	0.51	0.68

^†^: Multivariate model adjusted for age, sex, peptic ulcer history, ulcer bleeding history, *Helicobacter pylori* eradication rate, and comorbidities.

^‡^: Multivariate model adjusted for age, sex, peptic ulcer history, ulcer bleeding history, *Helicobacter pylori* eradication rate, comorbidities, and medications.

*OR* odds ratios, *N* number.

*P < 0.05.

**Table 3:** Associations between rosiglitazone use and gastric cancer risk in a population-based nested case–control study

**Variables**	**Rosiglitazone**						
	**Cases**		**Controls**		**Crude OR**	^**†**^**Adjusted OR**	^**‡**^**Adjusted OR**
	**N = 357**	**%**	**N = 1,428**	**%**			
Nonuse	323	90.5	1,217	85.2	1.00	1.00	1.00
Any use	34	9.5	211	14.8	0.61*	0.75	1.21
Recent use	10	2.8	25	1.8	1.51	1.50	1.88
Past use	24	6.7	186	13.0	0.49*	0.62*	0.93
Cumulative dosage							
< 260 DDDs	23	6.4	94	6.6	0.92	1.14	1.69
≥ 260 DDDs	11	3.1	117	8.2	0.35*	0.44*	0.79
Cumulative duration							
< 1 year	26	7.3	116	8.1	0.85	1.04	1.56
≥ 1 year	8	2.2	95	6.7	0.32*	0.40*	0.74

^†^: Multivariate model adjusted for age, sex, peptic ulcer history, ulcer bleeding history, *Helicobacter pylori* eradication rate, and comorbidities.

^‡^: Multivariate model adjusted for age, sex, peptic ulcer history, ulcer bleeding history, *Helicobacter pylori* eradication rate, comorbidities, and medications.

*OR* odds ratios, *N* number.

*P < 0.05.

A total of 9.5% of the patients and 14.8% of the controls had used some quantity of at least 2 prescriptions for rosiglitazone, as shown in Table 3. Any-use of rosiglitazone (OR = 0.61, P < 0.05) was associated with a decreased crude OR for gastric cancer. After adjusting for possible confounders, any-use of rosiglitazone (OR = 1.21, P *>* 0.05) had no significant association with decreasing gastric cancer. When rosiglitazone use was categorized by cumulative dosage, the crude OR was 0.92 (P *>* 0.05) for the group with cumulative rosiglitazone use < 260 DDDs, and was 0.35 (P < 0.05) for the group with cumulative rosiglitazone use ≥ 260 DDDs, as compared with non-use. After adjusting for possible confounders, no significant trend was observed toward decreasing gastric cancer risk with increasing cumulative doses ≥ 260 DDDs (OR = 0.79, P *>* 0.05). When rosiglitazone use was categorized by cumulative duration, the crude OR was 0.85 (P *>* 0.05) for the group with cumulative rosiglitazone use < 1 year, and was 0.32 (P < 0.05) for the group with cumulative rosiglitazone use ≥ 1 year compared with non-use. After adjusting for possible confounders no significant trend toward decreasing gastric cancer risk was noted with increasing cumulative duration ≥ 1 year (OR = 0.74, P *>* 0.05), as shown in Table 3.

## Discussion

Our current study is the first clinical epidemiological study to determine whether TZDs have a protective effect against gastric cancer. The results have demonstrated a null association between the effect of TZDs and gastric cancer in diabetic patients in Taiwan.

In our analysis, neither recent use nor past use affected the association with the overall risk of gastric cancer. However, increased mean daily dosages of pioglitazone ≥ 260 DDDs (OR = 0.70, P > 0.05) and rosiglitazone ≥ 260 DDDs (OR = 0.79, P > 0.05) were associated with a lower risk of gastric cancer after adjusting confounders, such as such as age, sex, peptic ulcer history, ulcer bleeding history, *H. pylori* eradication rate, comorbidities, and medications, which indicated a protective effect from gastric cancer occurrence with higher TZDs dosages, but this did not reach statistical significance. A similar trend was observed when the cumulative duration ≥ 1 year in pioglitazone (OR = 0.47 P < 0.05) and rosiglitazone (OR = 0.32 P < 0.05) were associated with a lower risk of gastric cancer. However, the risk reduction substantially diminished when adjusting for confounding factors; the statistical significance disappeared. This is not consistent with previous in vitro studies on TZDs, which showed antiproliferation and prodifferentiation effects [19,27,28].

In previous reports on the association between TZD use and gastric cancer in vitro or in vivo studies conducted by Leung et al. [27], the growth suppressive effect of high dosages of PGJ2 (10 uM) and ciglitazone (20 uM) were accompanied by apoptosis induction with a modest increase in DNA fragmentation. PPAR-γ ligands suppressed both in vitro and in vivo growth of gastric cancer, and may play a crucial role in cancer therapy and prevention [18,27]. These results also showed dose-dependent reduction in COX-2 mRNA expression after treatment with PPAR-γ ligand. In our study, we used a cumulative dosage (≥ 260 DDDs) and duration (≥ 1 year) to evaluate the effect of pioglitazone or rosiglitazone on the occurrence of gastric cancer in a clinical epidemiological study but showed a null association between TZDs and the occurrence of gastric cancer.

PPAR-γ is a ligand-dependent transcription factor involved in various processes, including inflammation and carcinogenesis. Several potential mechanisms have been proposed and investigated. The basis for therapeutic use as an anti-diabetic drugs is because the activation of PPAR-γ leads to improve insulin sensitivity and lower serum glucose during hyperglycemia. PPAR-γ, in combination with PPAR-γ ligands lowers the effects of interleukin-1 (IL-1)、IL-6、and tumor necrosis factor-α (TNF-α) [29]. In an in vitro study, PPAR-γ results in down-regulation of the expression of proinflammatory cytokines and inhibition of tumor cell growth [9]. It has been suggested that PPAR ligands are useful as anti-inflammatory drugs for inflammatory diseases. The antineoplastic effects are mediated by multiple pathways including suppression of COX-2, inhibition of the antiapoptotic B-cell lymphoma-2 (Bcl-2)/Bcl-extra-large (Bcl-XL) family and cyclin E1, and activation of p53 [27]. This activation of the PPAR ligand can suppress the activity of NF-κB [30]. The results of our study are not consistent with the assumed biological mechanism of TZDs, although how the mechanism of TZD use may decrease gastric cancer risk is not well understood or verified.

Konturek et al. and Slomiany et al. confirmed a direct link between *H. pylori* infection gastric cancer patients and overexpression of PPAR-γ and proinflammatory cytokines in such infected gastric mucosa [20,31]. Gupta et al. [32] showed that PPAR-γ ligands significantly attenuated *H. pylori*–induced apoptosis; this effect was reversed by co-treatment with a specific PPAR-γ antagonist. *H. pylori* infection is likely to become the first target in prevention strategies, particularly in high gastric cancer risk countries [33], such as Taiwan. In our study, we adjusted the potential confounders, such as the *H. pylori* eradication rate, peptic ulcer history, and ulcer bleeding history, to determine the association of TZDs and the occurrence of gastric cancer. We also controlled other confounding factors, include comorbidities such as CAD, CVD, CLD, COPD, CKD, GERD, and EE, and medications, such as metformin, sulfonylurea, glucosidase inhibitors, meglitinides, DPP-4 inhibitors, insulin, statins, ARBs, ACE inhibitors, aspirin, NSAIDs, and COX-2-specific inhibitors to minimize the limitations of nested case–control studies.

One of the strengths of our study is the use of a computerized database, which is population-based, and is highly representative. TZDs are available only by prescription. Because TZD-use data were obtained from a historical database that collected all prescription information before the date of gastric cancer diagnosis, the recall bias for TZDs use was avoided. Second, the primary exposure of interest was the use of pioglitazone or rosiglitazone, which entered Taiwan’s market in June 2001 and March 2000, respectively, and is covered within our patient enrollment in the database.

Caution should be taken in extrapolating our results to other populations. Certain limitations in our study exist. First, we did not obtain the *H. pylori* status and also had no information on whether the patients ever received *H. pylori* eradication before 1997. *H. pylori* infection is associated with gastric cancer development, and early eradication of *H. pylori* can reduce the risk of gastric cancer [34]. Therefore, we adjusted confounders, such as the *H. pylori* eradication rate, to minimize these limitations. Second, a lack of patient drug adherence data was noted in the database for DM patients using pioglitazone or rosiglitazone; therefore, the drug effects may have been overestimated. Third, we did not obtain life-style risk factors, such as smoking, obesity, physical activity, or family history of gastric cancer, which may affect the association of DM to gastric cancer. Fourth, the association of TZDs to gastric cancer might be confounded by the severity of DM and the patients’ levels of glucose control; however, we lacked this data. We could not examine whether TZDs had a better glucose-lowering effect compared with non-TZD use. Fifth, we did not have the socioeconomic status of our patients. However, confounding by socioeconomic status is minimal because the NHI system in Taiwan has comprehensive coverage, and allows patients to visit any clinic or hospital freely without referral by a general practitioner. People in Taiwan have few barriers to medical service in terms of accessibility and cost [35]. Sixth, because of the relatively small numbers of cases; therefore, well-designed epidemiologic studies that examine large computerized health database may provide useful information to confirm the association of TZDs with gastric cancer prevention. Because the number of cases with higher cumulative dosages (≥ 260 DDDs) was relatively small, we were unable to examine the higher concentration effect of TZDs on gastric cancer occurrence. Moreover, as our average cumulative treatment duration of TZDs was also relatively shorter, we were unable to examine the long-term effects of TZDs on gastric cancer occurrence. Seventh, we observed differential associations between pioglitazone and rosiglitazone with the occurrence of gastric cancer. Despite numerous in vitro and animal studies supporting the protective effects of TZDs, we were unable to identify the exact underlying physiological pathways that resulted in reduced cancer risk. Rosiglitazone has PPAR-γ activity but pioglitazone has both PPAR-α and PPAR-γ activities. The medication for cancer protection and inhibition through dependent and independent pathways may differ between pioglitazone and rosiglitazone [28,36]. Therefore, we separated pioglitazone and rosiglitazone to evaluate the risk of occurrence of gastric cancer.

In summary, our study indicates that TZDs cannot suppress gastric cancer occurrence. This study is conducted using data from the NHI database, which is nationwide-based and highly representative. The exact mechanism must be further elucidated, and the role of PPAR-γ ligands in chemoprevention and therapy of human gastric cancer warrants further evaluation. Additional large prospective studies must be conducted to elucidate this issue.

## Conclusions

In conclusion, our results did not provide evidence to support that TZD derivatives in DM patients reduces gastric cancer occurrence.

## Competing interests

The authors declare that they have no competing interests.

## Authors’ contributions

H-YH Ph.D. was responsible for performing database management, data analysis and statistic testing. S-SC M.D., MPH assisted with the design and implementation of the study. The manuscript was written mainly by S-SC. H-YH and S-SC contributed equally to this study. Both authors read and approved the final manuscript.

## Pre-publication history

The pre-publication history for this paper can be accessed here:

http://www.biomedcentral.com/1471-2407/13/420/prepub
